# Use of DIAGNOdent and VistaProof in diagnostic of Pre-Cavitated Caries Lesions—A Systematic Review and Meta-Analysis

**DOI:** 10.3390/jcm9010020

**Published:** 2019-12-19

**Authors:** José Enrique Iranzo-Cortés, José María Montiel-Company, Teresa Almerich-Torres, Carlos Bellot-Arcís, José Manuel Almerich-Silla

**Affiliations:** Stomatology Department, University of Valencia, 46010 Valencia, Spain; j.enrique.iranzo@uv.es (J.E.I.); Teresa.Almerich@uv.es (T.A.); Carlos.Bellot@uv.es (C.B.-A.); jose.m.almerich@uv.es (J.M.A.S.)

**Keywords:** caries diagnosis, laser fluorescence, incipient caries, DIAGNOdent, VistaProof

## Abstract

**Objective:** To analyse the published evidence of the validity of DIAGNDOdent and VistaProof in diagnosing carious depths in pre-cavitated lesions. **Material and methods:** A systematic review was carried out after identifying a total of 184 articles, including 27 concerning the qualitative review and a subsequent meta-analysis. The quality of the studies was evaluated by using the QUADAS-2 tool. **Results:** For DIAGNOdent, the sensitivity value was 0.77, the specificity value was 0.75 and AUC was 0.81 for the global meta-analyses. In relation to subgroups, the values estimated 0.85, 0.76 and 0.86, respectively, for the in vivo group and 0.71, 0.75 and 0.83 for the in vitro group. For VistaProof, sensitivity was 0.81, specificity 0.75 and AUC had a value of 0.80 in the global meta-analysis. For the subgroups, these were considered at 0.75, 0.81 and 0.89, respectively, for the in vivo group and 0.91, 0.74 and 0.76 for the in vitro group. Neither case presented publication bias when analysing the funnel plot, the classic fail-safe number and Egger’s intercept. **Conclusion:** Both VistaProof and DIAGNOdent are valid as they offer a moderate to high diagnostic effectiveness for dentine depth in pre-cavitated lesions.

## 1. Introduction

The term “dental caries” is used to describe the results (signs and symptoms) of a chemical dissolution located on the dental surface caused by metabolic processes taking place in the biofilm covering the treated area. Thus, dental caries is localised destruction of the tooth and it is often described as chronic disease, or a very slow process present in most individuals. It is of multifactorial origin and is one of the most prevalent conditions among the public [1].

In a continuous process, as is the case of caries, different stages in the disease may be considered in determining the presence of lesions. The value of the caries prevalence may be quite different according to the onset or the stage of the disease considered in its diagnosis. To date, the diagnostic threshold has been determined by the detection limits inherent in traditional diagnostic methods. Today, the low prevalence of observed dental caries, in many countries, has led to researchers’ added interest in obtaining more refined diagnostic tools which can detect caries lesions before they become visible to the naked eye [2].

Changes in caries presentation patterns, apart from pointing the way towards improving visual diagnosis, have led to the necessary creation or use of reliable diagnostic methods that avoid the generation of false positives or negatives which lead to diagnostic error [2]. A major consideration is that the diagnostic method should be reproducible given that otherwise, the long-term monitoring of lesions would be ruled out. In addition to caries diagnosis, it is essential to quantify the lesion, both for clinical decision-making and to monitor lesions to gauge their progress over time.

In recent years, different diagnostic methods for incipient caries have been developed further and those based on fluorescence have made major advances. The demineralization of hard dental tissue leads to the gradual loss of its natural fluorescent properties and the appearance of chemical substances called porphyrins within the lesion. These changes in fluorescence may be quantified and measured, hence offering a non-invasive diagnostic method. By using a light-emitting diode (LED) the fluorescence of the dental surfaces is stimulated, as are the porphyrins. The fluorescence received is captured by a receptor; information is processed and analysed and different fluorescence values are generated as a function of the depth of the lesion [3,4].

There are various fluorescence-based methods, of which the most commonly studied are KAVO^®^ DIAGNOdent and VistaProof by Durr Dental^®^. In the former, we used a laser-light instrument emitting a 655 nm wavelength that stimulates the fluorescence inside of the lesions and which quantifies a value that ranges between 0 and 99 [3]. On the other hand, VistaProof offers an intraoral camera which emits a 405 nm light wavelength and takes images that are computer-processed; the resulting mapping of the lesion is produced according to its depth [5].

The objective of this systematic review was to analyse the published evidence on the validity of KAVO^®^ DIAGNDOdent and VistaProof by Durr Dental^®^ to diagnose caries’ depth in pre-cavitated lesions.

## 2. Experimental Section

### 2.1. Materials and Methods

In satisfying the objectives of this study, we pose the following “PICOS” question: “Are the diagnostic methods based on fluorescence (Intervention) valid (Outcome) in comparison with the histologic diagnosis (Comparison) in teeth affected by pre-cavitated lesions (Population)?”. The study type analysed are diagnostic validity studies (S).

### 2.2. Criteria for the Selection of Studies

The selection criteria were based on studies that used both VistaProof as well as DIAGNOdent and which examined the diagnostic validity of both methods, without any date limits, published in English and furthermore, where use was made of the ICDAS criteria for the visual diagnosis of caries. Those that were excluded were studies that used diagnostic instruments for other purposes; also excluded were systematic reviews.

### 2.3. Article Search and Selection Strategies

To identify the articles, regardless of language, an electronic search in databases was carried out in PubMed, Scopus, Embase and Web of Science. The search was carried out during April 2018.

The search strategy was based on the combination of the following terms: “laser fluorescence” OR “VistaProof” OR “DIAGNOdent” AND “caries” AND “ICDAS”. The search equation for PubMed was ((“lasers”[MeSH Terms] OR “lasers”[All Fields] OR “laser”[All Fields]) AND (“fluorescence”[MeSH Terms] OR “fluorescence”[All Fields])) OR vistaproof[All Fields] OR diagnodent[All Fields] AND (“dental caries”[MeSH Terms] OR (“dental”[All Fields] AND “caries”[All Fields]) OR “dental caries”[All Fields] OR “caries”[All Fields]) AND ICDAS[All Fields]. The equation for Scopus was (TITLE-ABS-KEY (laser AND fluorescence) OR TITLE-ABS-KEY (vistaproof) OR TITLE-ABS-KEY (diagnodent) AND TITLE-ABS-KEY (caries) AND TITLE-ABS-KEY (icdas)). For Embase, the equation used was ‘laser fluorescence device’ AND dental AND caries AND icdas. Finally, to use Web of Science the equation applied was TOPIC: (caries) AND TOPIC: (vistaproof) OR TOPIC:(diagnodent) AND TOPIC:(ICDAS).

Two qualified reviewers (JEI-C and JMM-C) selected the articles independently. In case of disagreement, a third reviewer, (JMA-S) decided if the study was to be included or not. A Kappa value of 0.92 was obtained to determine inter-reviewer reliability. The initial selection was carried out by reading the article titles and abstracts. Whenever the information was found to be insufficient, the whole article was read before making a final decision.

### 2.4. Data Extraction

For each of the articles selected, authors’ names were registered, as were year of publication, number of examiners, sample size, whether on primary or permanent dentition, instrument used (VistaProof or DIAGNOdent), validity (sensitivity, specificity and area under the curve), and the Gold Standard reference test used.

### 2.5. Study Quality Analysis

The quality of the studies was established by applying the QUADAS-2 tool [6] which evaluates bias probabilities in carrying out the studies, in the selection of patients, individuals, the index test used, the reference standard test, the flow and the timing. Also considered in a second section is the question of suitability and applicability of the results obtained in carrying out other studies. For each of the items, a low, uncertain or high level of bias probability is established.

### 2.6. Quantitative Analysis of the Studies (Meta-Analysis)

Three variables of effect size were estimated: sensitivity, specificity and AUC, both for DIAGNOdent and VistaProof, based on studies included in the meta-analysis. In the case of a given study presenting data from more than one examiner in relation to validity, only data obtained by the most experienced examiner were considered. In the case of presenting different results on the histologic level, only results referring to D3 were considered. Similarly, even though new cut-off points were calculated, the results that strictly followed the specifications of the manufacturer were considered.

The studies included in the meta-analysis were combined according to the random effect model. The estimation was considered significant when the Z value was <0.05. The heterogeneity was assessed by using *I*^2^ and the Q-test. The *I*^2^ parameter indicates that there is a high degree of heterogeneity when the value is greater than 75%, moderate if it falls between 74% and 50% and low if it is between 25% and 49%. However, when the Q statistics present a *p*-value < 0.1, it is considered that heterogeneity exists. To identify possible sources of heterogeneity, stratified meta-analyses by subgroups were carried; these were differentiated according to whether the studies were in vivo or in vitro. The sensitivity analysis took place according to the One Study Removed Method which evaluates the stability of estimation of effect size which is obtained each time a study is eliminated from the meta-analysis.

The publication bias was analysed using 3 methods: the classic fail-safe number, Egger’s regression intercept and the funnel plot. The first of these estimates the number of studies which are statistically significant, and which would be necessary so that a meta-analysis with a significant result (*p* < 0.05) shall no longer be deemed significant. Egger’s intercept considers that it is significant, and hence there is no publication bias, when the *p*-value is <0.1. Finally, the funnel plot values the distribution symmetry of the studies represented in a graph with two axes: The Y axis (standard error) and the X axis (logit event rate). The meta-analysis was carried out with Comprehensive Meta-analysis v3 software. (Biostat).

## 3. Results

### 3.1. Flow Diagram

**Figure 1:**
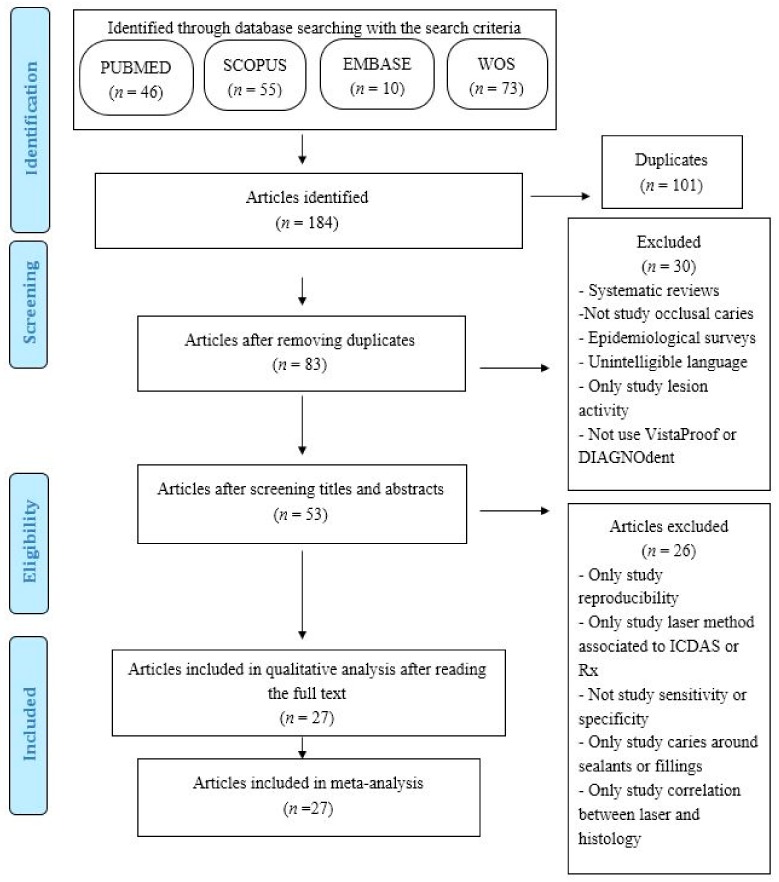
Flow diagram.

Applying the search criteria resulted in the identification of 184 articles (46 in PubMed, 55 in Scopus, 10 in Embase and 73 in Web of Science. After eliminating 101 duplicated articles, we were left with 83, of which 30 were subsequently eliminated after reading the title and y abstract. A total of 26 articles were rejected after a complete reading since they did not fit in with the objective of the study. Finally, 27 articles were suitable to be included in the systematic review and subsequent meta-analysis (Figure 1)

### 3.2. The Qualitative Analysis

Of the 27 articles included, 23 used DIAGNOdent [3,4,7,8,9,10,11,12,13,14,15,16,17,18,19,20,21,22,23,24,25,26,27], studying sensitivity, specificity and AUC in vitro in 16 of them [4,7,8,9,10,11,13,14,15,16,17,20,23,24,25,26] while nine were in vivo [3,12,16,18,19,21,22,26,27]; in two cases, however, (Theocharopoulou 2015 [21] and Peycheva 2016 [24]) carried out the study in vivo and in vitro. For VistaProof, 14 articles were reviewed [4,5,7,8,10,12,14,15,19,23,27,28,29,30], of which 10 were in vitro [4,7,8,10,14,15,23,28,29,30] and 4 were done in vivo [5,12,19,27]. Most of the studies presented a large sample size of approximately 100, even though they range from 32 in the study by Melo 2015 [19] to the high figure of 433 in Rechmann’s study 2012 [3]. The results are presented in Table 1.

**Figure 2:**
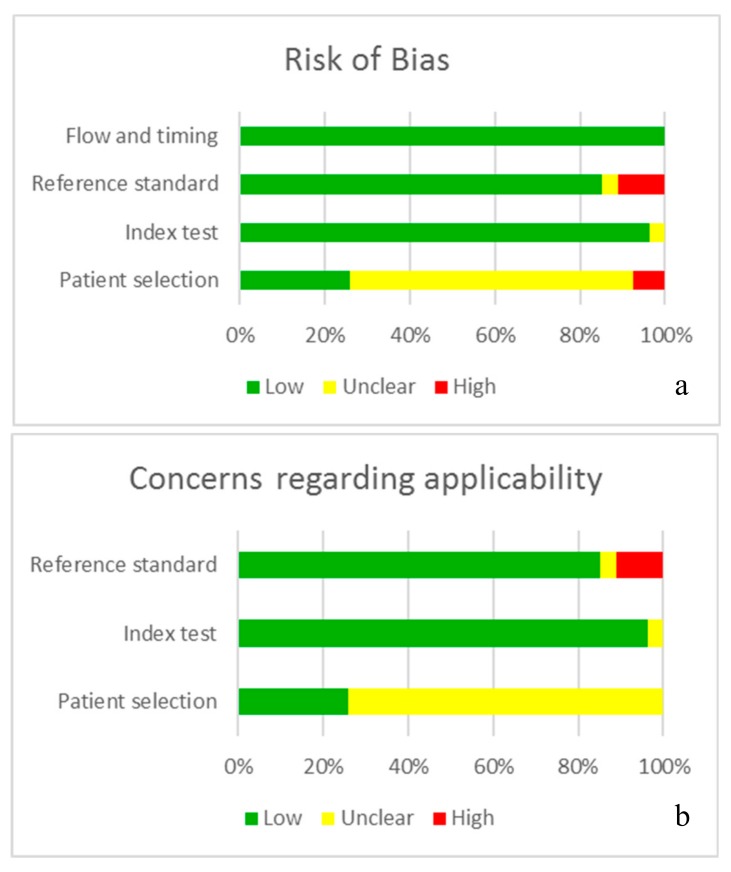
Information is presented in graph form; the QUADAS-2 tool indicates the quality of the studies, analysing the probability of bias (**a**) and the concern about applicability (**b**).

The quality of study analysis is reflected in Table 2 as well as in Figure 2, which represent the risk of bias (Figure 2a) and the concerns about applicability(Figure 2b) of the articles analysed by QUADAS-2. Most of the articles were of good quality, and probability of bias in the selection of the sample was possibly the most likely form of bias in the studies and the main concern regarding applicability.

### 3.3. Quantitative Analysis of Studies Referring to DIAGNOdent

#### 3.3.1. Sensitivity Study

**Figure 3:**
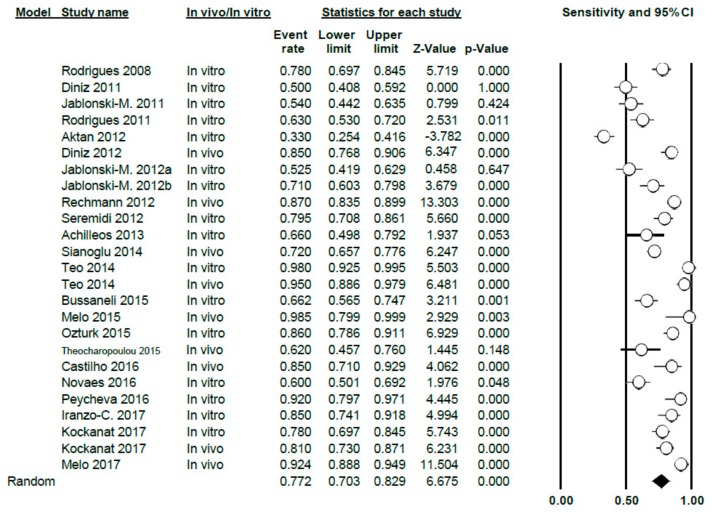
Forest Plot of sensitivity for DIAGNOdent.

A total of 23 studies on the sensitivity of DIAGNOdent in the meta-analysis, presenting a Q-value = 331.91 (*p* < 0.005) and *I*^2^ = 92.77, indicating a high degree of heterogeneity. Using the random effects model for the combination of the studies, a sensitivity level of 0.77 was estimated, with a confidence level of 95% between 0.70 and 0.83 (Figure 3).

**Figure 4:**
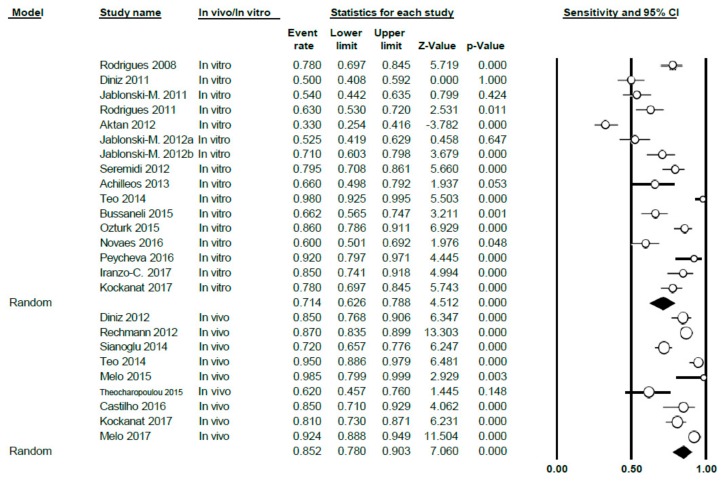
Forest Plot by sub-groups in vivo/in vitro of sensitivity for DIAGNOdent.

The meta-analysis was carried out according to sub-groups, differentiating between studies in vitro (16 studies) and in vivo (9 studies); the sensitivity value obtained was 0.71 (0.63–0.79) for the in vitro group, with high heterogeneity (Q = 164.5; *p* < 0.005; *I*^2^ = 90.9) and also 0.85 (0.78–0.90) for the in vivo group; heterogeneity was also high in this case (Q = 63.81; *p* < 0.005; *I*^2^ = 87.46) (Figure 4).

#### 3.3.2. Study of Specificity

**Figure 5:**
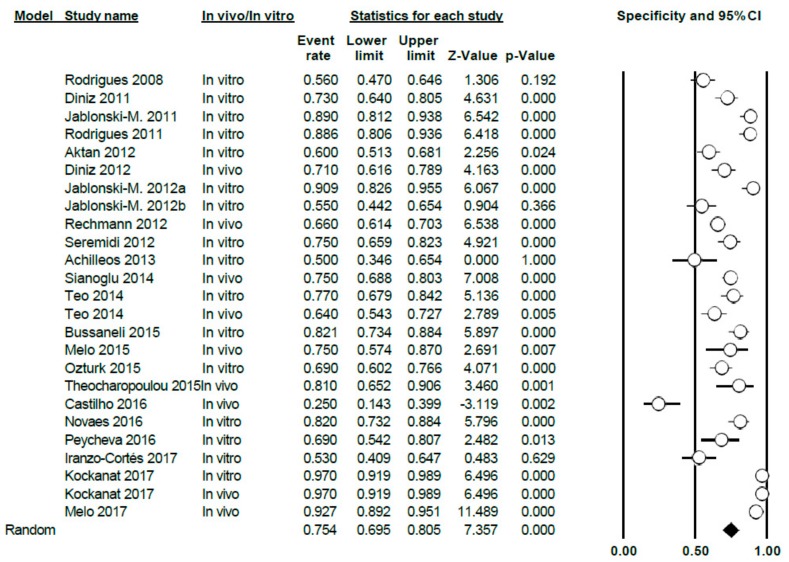
Forest Plot for DIAGNOdent specificity.

The specificity value of DIAGNOdent was 0.75 (0.69–0.81), using the random effects model; high heterogeneity between the studies was shown (Q = 247.72; *p* < 0.005; *I*^2^ = 90.31) (Figure 5).

**Figure 6:**
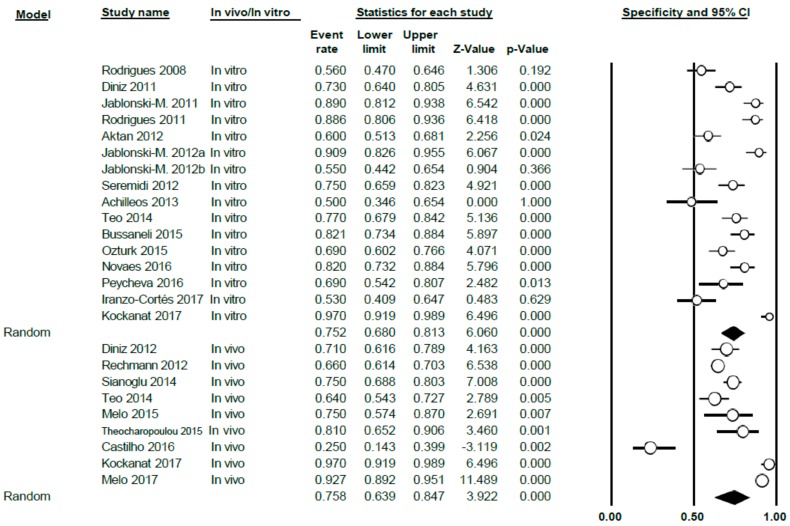
Forest Plot by sub-groups in vivo/in vitro for DIAGNOdent specificity.

Differentiating between sub-groups, between in vitro and in vivo studies, the in vitro specificity level was estimated at 0.75 (0.68–0.81); for in vivo studies, the estimated specificity was 0.76 (0.64–0.85). In both cases, heterogeneity continued to be high (Q = 125.85; *p* < 0.005; *I*^2^ = 88.81 in vitro and Q = 121.64; *p* < 0.005; *I*^2^ = 93.42 in vivo, respectively) (Figure 6).

#### 3.3.3. AUC Study

**Figure 7:**
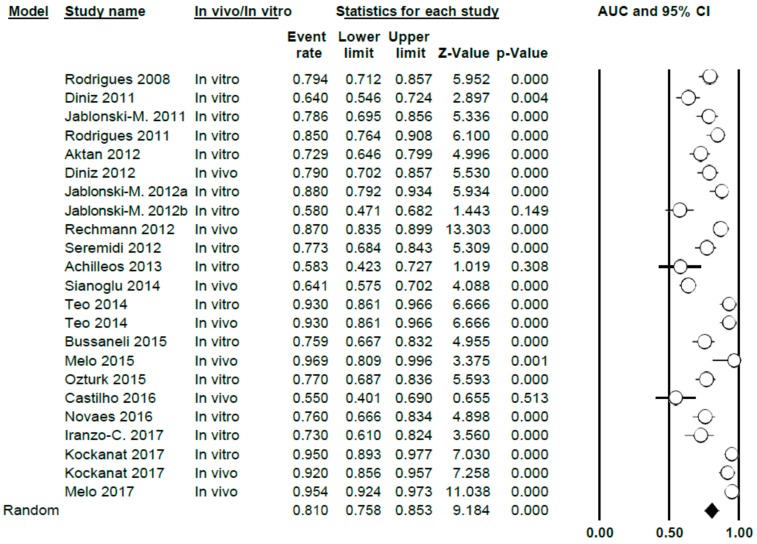
AUC Forest Plot for AUC for DIAGNOdent.

The AUC value for DIAGNOdent was 0.81 (0.76–0.85), thus presenting a high level of heterogeneity between the studies (Q = 201.7; *p* < 0.005; *I*^2^ = 89.1) (Figure 7). This meta-analysis excluded two studies (Rechmann, 2012 [3] and Theocharopoulou, 2015 [21]) as AUC was not indicated in their results.

**Figure 8:**
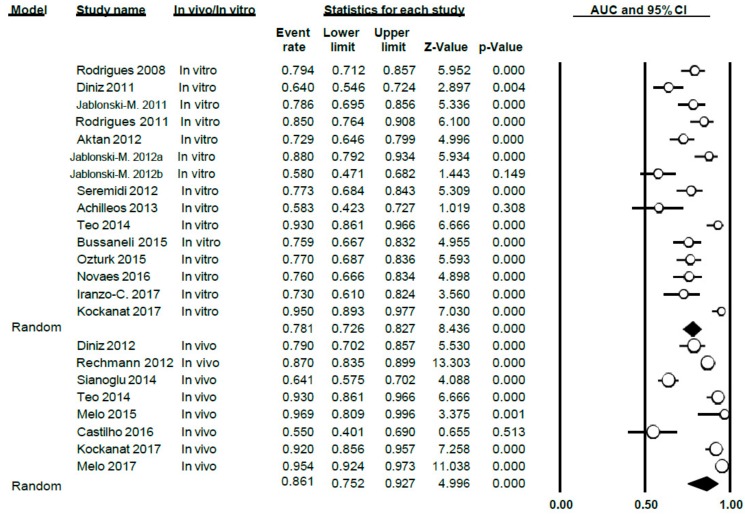
AUC Forest Plot according to in vivo/in vitro sub-groups for DIAGNOdent.

When differentiating according to sub-groups in the meta-analysis, the AUC values obtained were 0.78 (0.73–0.83) and 0.86 (0.75–0.93), in vitro and in vivo, respectively, with a high degree of heterogeneity in all cases (Q = 73.05; *p* < 0.005; *I*^2^ = 80.83 in vitro and Q = 119.02; *p* < 0.005; *I*^2^ = 94.20 for in vivo) (Figure 8)

#### 3.3.4. Publication Bias

**Figure 9:**
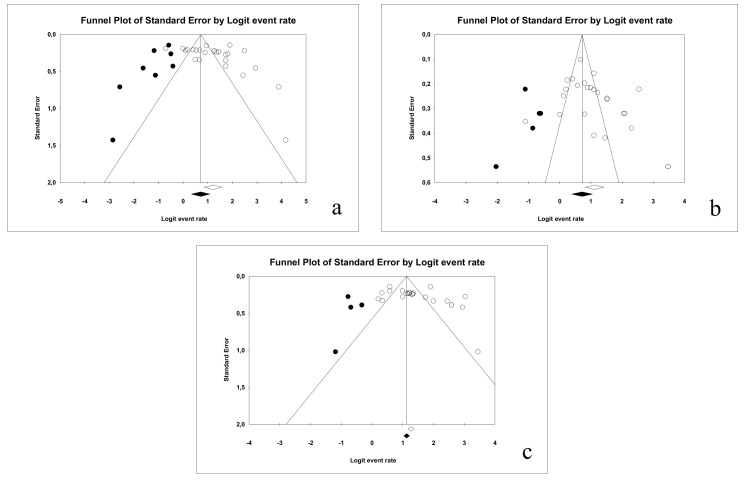
Funnel plot for the publication bias in the case of: sensitivity (**a**), specificity (**b**) and AUC (**c**) for DIAGNOdent.

In relation to sensitivity, a classic fail-safe number of 3032 was obtained; Egger’s intercept was 3.35 with a *p*-value of 0.12 and a standard error of de 2.05; from these results, it can be deduced that there is no publication bias. The funnel plot can be observed in Figure 9a.

In relation to specificity, a classic fail-safe number of 2841 was obtained and Egger’s intercept was 3.27 (two-tail *p*-value was 0.054 and standard error was 1.62); hence, publication bias is ruled out. The funnel plot is illustrated in Figure 9b.

Finally, for the area under the curve, a classic fail-safe value of 3954 was obtained, while Egger’s intercept was 3.63 (*p*-value: 0.061 and standard error was 1.83), indicating no publication bias in this case. The resulting funnel plot is illustrated in Figure 9c.

### 3.4. Results for VistaProof

#### 3.4.1. Study of Sensitivity

**Figure 10:**
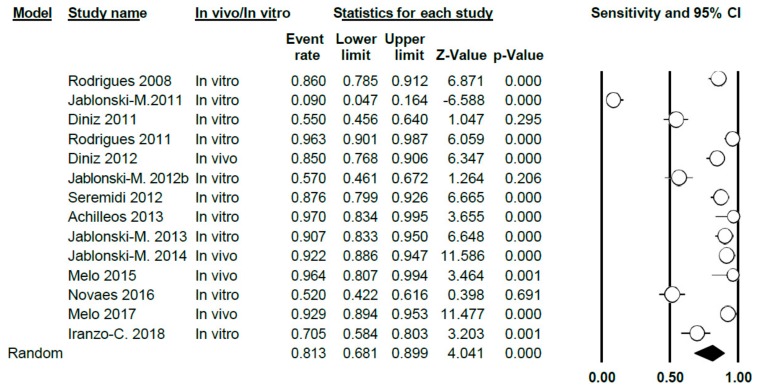
Forest Plot of VistaProof sensitivity.

A total of 13 studies on the specificity of VistaProof in this meta-analysis; Q-value = 300.48 (*p* < 0.005) and *I*^2^ = 95.67, indicating a high degree of heterogeneity. Using the random effects model, sensitivity was estimated to be 0.81 (0.68–0.90) (Figure 10).

**Figure 11:**
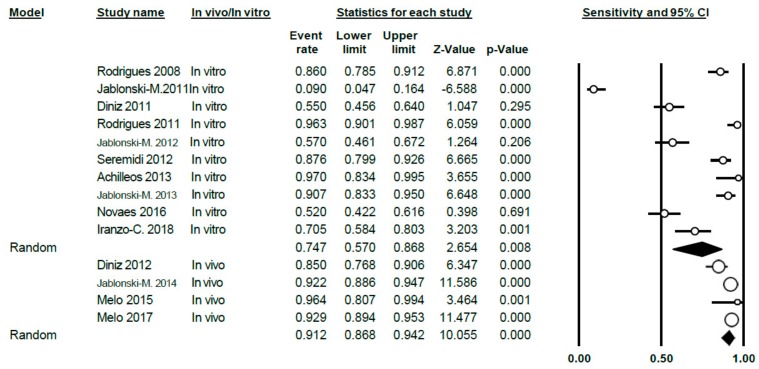
Forest Plot according to in vivo/in vitro subgroups for sensitivity when using VistaProof.

In carrying out the meta-analysis differentiating between in vitro studies (9 in total) and in vivo ones (four studies), a sensitivity value of 0.75 was obtained (0.57–0.87) for the in vitro group and showing a high degree of heterogeneity (Q = 182.23; *p* < 0.005; *I*^2^ = 95.06) and the value was 0.91 (0.87–0.94) for the in vivo group, respectively. There was no heterogeneity in this case (Q = 7.34; *p* = 0.062; *I*^2^ = 59.12) (Figure 11).

#### 3.4.2. Study of Specificity

**Figure 12:**
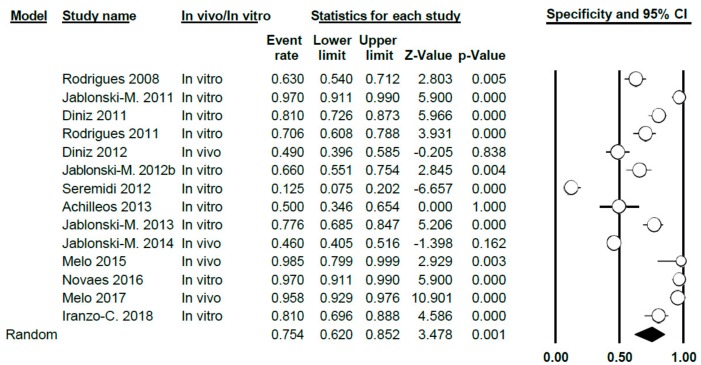
Forest Plot for specificity when using VistaProof.

For VistaProof, the specificity value was 0.75, with a 95% confidence interval ranging between 0.62 and 0.85. The random effects model was used, given the heterogeneity between the studies (Q = 292.05; *p* < 0.005; *I*^2^ = 95.55) (Figure 12).

**Figure 13:**
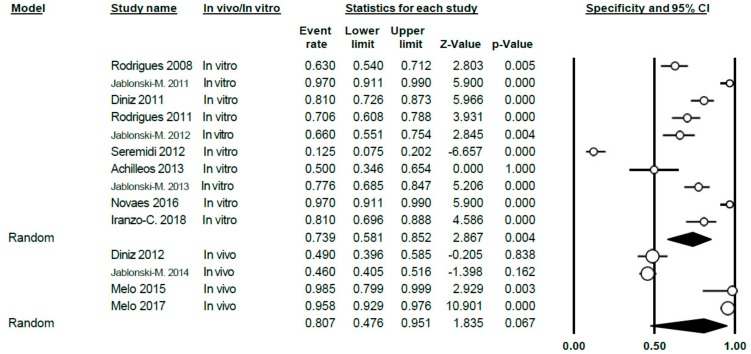
Forest Plot for in vivo/in vitro sub-groups; sensitivity when using VistaProof.

Differentiating between in vitro and in vivo studies, the specificity value in vitro was 0.74 (0.58–0.85), with high heterogeneity between studies (Q = 152.44; *p* < 0.005; *I*^2^ = 94.09). For in vivo studies, the specificity value was 0.81 (0.48–0.95), again, this case presenting a high heterogeneity (Q = 123.15; *p* < 0.005; *I*^2^ = 97.56) (Figure 13).

#### 3.4.3. AUC Study

**Figure 14:**
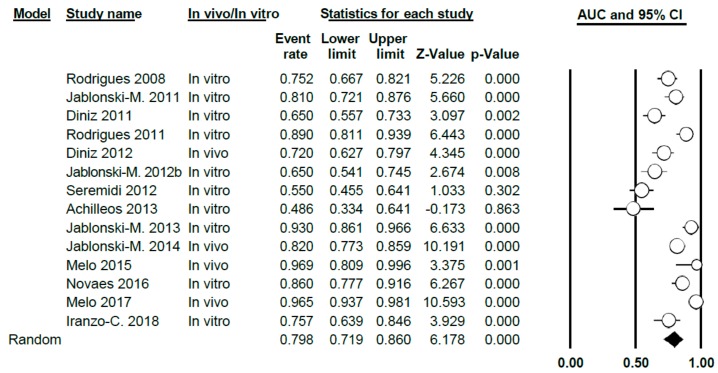
Forest Plot for AUC when using VistaProof.

The AUC value for VistaProof was 0.80 (0.72–0.86), applying the random effects model, given the heterogeneity existing between studies (Q = 138.36; *p* < 0.005; *I*^2^ = 90.60) (Figure 14).

**Figure 15:**
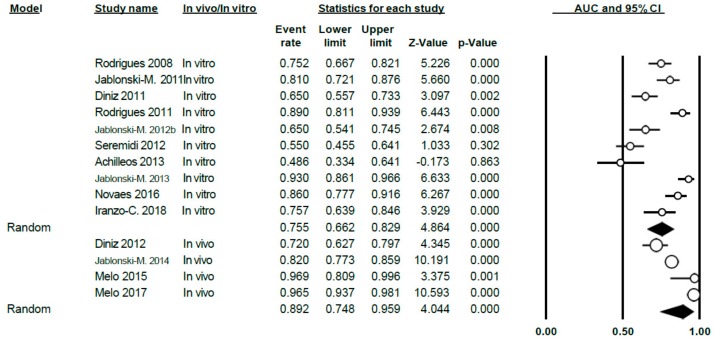
Forest Plot in vivo/in vitro sub-groups for AUC when using VistaProof.

Differentiating between in vitro and in vivo, the AUC value was 0.76 (0.66–0.83) for the in vitro group; for the in vivo it was 0.89 (0.75–0.96). In both cases, heterogeneity was demonstrated (Q = 72.93; *p* < 0.005; *I*^2^ = 87.6 for the in vitro group and Q = 42.70; *p* < 0.005; *I*^2^ = 92.98 for the in vivo group) (Figure 15).

#### 3.4.4. Publication Bias 

The same parameters were studied as in the DIAGNOdent studies; they were applied in the areas of sensitivity, specificity and AUC. No publication bias was found in each of these three cases.

**Figure 16:**
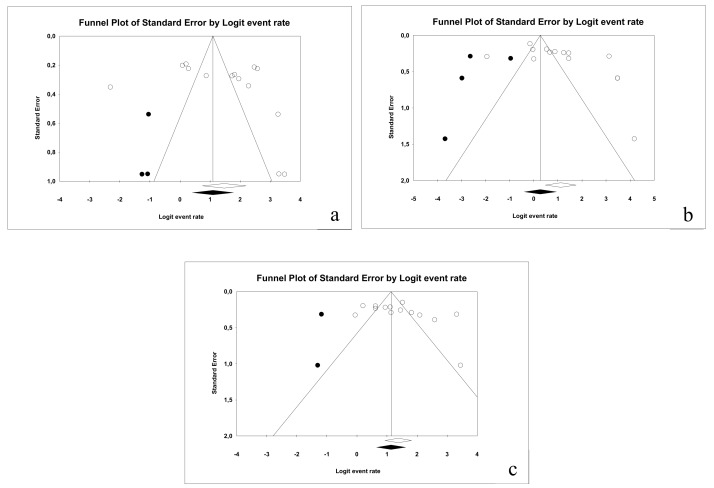
Funnel plot for the publication bias study in the cases of sensitivity (**a**), specificity (**b**) and AUC (**c**).

As for sensitivity when using VistaProof, a classic fail-safe number of 890 was obtained, while Egger’s intercept was established at 3.24 with a two-tailed *p*-value of 0.41 and a standard error value of 3.80. Figure 16a illustrates the funnel plot.

In relation to specificity, a classic fail-safe number of 366 was obtained; in this case, Egger’s intercept was 5.53 (*p* = 0.06 and standard error = 2.68). The funnel plot is illustrated in Figure 16b.

Finally, for AUC a classic fail-safe number of 1100 was obtained. Egger’s intercept was 3.83 (*p* = 0.19 and standard error = 2.74). The funnel plot is illustrated in Figure 16c.

All the results for sensitivity, specificity and AUC obtained in the meta-analysis for DIAGNOdent and VistaProof are showed in Table 3.

## 4. Discussion

The reduction in the prevalence of cavitated caries has made it necessary to use instruments that will improve the diagnosis of pre-cavitated lesions [31]. A wide range of instruments have been designed with this in mind; in this review and meta-analysis, two based on fluorescence were examined: DIAGNOdent and VistaProof.

Both instruments have been evaluated in many studies, both in vivo and in vitro, however, considerable discrepancy have appeared in the results. When analysing the sensitivity value of DIAGNOdent, most studies offer positive results, with moderate to high values; the value obtained in the meta-analysis, in which 23 articles were included, is moderate (0.77). Mention must be made of the study by Aktan et al. 2012 [11]; this work was the only one that determined a sensitivity below 0.50. The authors attribute this value to the relative lack of experience by the examiners, to the sample type selected for the study or to the kind of solution used to store the teeth.

Regarding specificity, the value obtained for DIAGNOdent in the meta-analysis was 0.75, a moderate value. The individual results of each study show a moderate to high specificity, except in the study by Castilho et al. 2016 [22], which generated a specificity value of 0.25. The authors justified this result due to the low number of teeth in the study with histologic lesions that reached the D3 threshold.

This low specificity in the study by Castilho et al. 2016 [22] resulted in the AUC obtaining the smallest value among the studies included in the systematic review (0.55), even though other studies such as those by Achilleos et al. 2013 [4] or Jablonski-Momeni et al. 2012b [14] also generated similar results (0.58, in both). In spite of this, the remainder of results gave AUC values that ranged between moderate and high, the maximum being 0.97, obtained by Melo et al. in 2015 [19]; nonetheless, it must be borne in mind that this study was carried out with a small sample (32 teeth). The value obtained in the meta-analysis for DIAGNOdent was 0.81.

The analysis that used the one-study removed method showed that the value obtained in the meta-analysis was not significantly affected by the inclusion of these studies that presented quite mixed results in terms of the sensitivity, specificity or area below the curve (Supplementary Figures S1–S3).

Regarding VistaProof, estimated sensitivity in the meta-analysis was 0.82. The majority of the 14 studies included in the meta-analysis showed a sensitivity that ranged between moderate and high, with the exception of the results obtained by Jablonski-Momeni et al. in 2011a [28].

The results obtained for VistaProof in relation to specificity presented greater variability, between 0.13 (Seremidi et al. 2012 [15]) and 0.97 (Jablonski-Momeni et al. 2011a [28] and Novaes et al. 2016 [23]), although the majority show specificity results that fall between moderate and high. The value in this case was 0.75.

As for AUC in the case of VistaProof, a value of 0.80, was obtained and therefore this instrument offers good diagnostic precision. The results of the studies included were mixed, ranging between low and high values. The study by Achilleos et al. dating from 2013 was the only one that presented an unacceptable area below the curve, with a value below 0.50 [4]. The authors of this study attributed this result to the fact that the majority of lesions studied in their sample were enamel lesions (21 out of 38, while there were only 2 out of 38 with healthy surfaces; dentine lesions were found in15 out of 38 cases), hence leading to poorer results in relation to other studies with higher numbers of dentine lesions and where the instrument was found to be more precise. It must be borne in mind that for our meta-analysis, the results obtained from the histologic results observed in the D3 cut-off point (dentine) have always been considered. Furthermore, the study entails a small sample (only 38 surfaces studied) and therefore, the results are less representative due to reduced variation in lesion depth and presence of healthy teeth).

In the case of VistaProof, the sensitivity study using the One-Study Removed Method did not show that the elimination of one of the studies might have affected the final value of none of the three meta-analyses that were carried out (sensitivity, specificity or AUC) (Supplementary Figures S4–S6).

The high heterogeneity detected among the studies included in the different meta-analyses may pose a limitation in the study. The authors thought that as the exam conditions differ between in vivo and in vitro studies, maybe it could be a source of heterogeneity. To solve the problem of the high degree of heterogeneity, analyses were carried out for sub-groups, differentiating between in vivo and in vitro studies, however, no significant differences were found. Other possible sources that we may consider may include type of dentition, as the tooth enamel of temporary teeth and permanent teeth have certain differences in terms of structure and composition. However, since in only four of the 27 studies included in the qualitative analysis was temporary dentition considered, the sub-group analysis was ruled out.

We must bear in mind that VistaProof, as well as DIAGNOdent are instruments that make use of fluorescence for the depth diagnosis of a lesion and that necessarily requires major standardisation of room lighting conditions and the position of the instrument regarding the lesion with respect to its manipulation by three examiners, an aspect which accounts for a major source of heterogeneity. In fact, studies which present different examiners show different results even though the same instrument may be used on the same sample [14,26,28]. To control this possible source of bias, our methodology has ensured that the data came from the most experienced examiner. The measurements carried out in the various studies are standardised per se but there is no common standardisation for the different studies that go beyond the manufacturer’s instructions.

To avoid publication bias, our meta-analysis included a systematic search of the key words in four different databases. The bias was analysed by using a funnel plot and Egger’s regression intercept; the result was absence of bias.

The results indicate two instruments with validity in the diagnosis of pre-cavitated lesion depths, without there being great differences between them; this finding confirms the results obtained in most studies included. Regarding sensitivity and specificity, both offer moderate to high results for both instruments; furthermore, the area under the curve in both cases present good diagnostic precision. These diagnostic instruments should be considered as complementary to any visual diagnosis.

Early stage caries diagnosis is difficult to achieve. Digital radiography and laser-fluorescence methods have been developed to help practitioners diagnosing non cavitated lesions. The main advantage of using these diagnostic methods is that they allow to avoid employing X-ray methods, so patient receives no radiations for the diagnosis [26]. Some studies [25,30] also show that they improve the accuracy of visual diagnosis. The validity of the visual diagnosis has been compared with DIAGNOdent [4,7,9,12,13,16,18,20,22,24,25,26] and with VistaProof [4,7,12,30], but few studies have analysed the combination of both methods as complementary to visual diagnosis. Iranzo-Cortés et al. [25,30] showed that these possible combinations increase the sensitivity values but decrease the question of specificity and the area under the curve, though not to a significant degree with respect to visual diagnosis only.

## 5. Conclusions

After analysing the available evidence on laser fluorescence methods in this systematic review, we can confidently affirm that VistaProof and DIAGNOdent are valid and offer a diagnostic efficacy which is moderate in the diagnosis of dentine depth in pre-cavitated lesions.

## Figures and Tables

**Table 1:** Results of the studies included in the review. Se:Sensitivity; Sp:Specifity; Az: Area Under ROC curve; the number indicates the examiner. Pri: Primary tooth; Per: Permanent tooth; D1: caries limited to enamel in histology; D3: caries in dentin in histology. * Histology, Rx o ICDAS when classified as sound by visual criteria (ICDAS = 0).

Study	Examiners	*n*	In Vivo/In Vitro	Dentition	VistaProof Results	DIAGNOdent Results		Reference Test
Rodrigues 2008 [7]	2	119	In vitro	Per	D3: Se:0.86;Sp:0.63;Az:0.752	D3:Se:0.78;Sp:0.56;Az:0.794		Histology
Diniz 2011 [8]	2	110	In vitro	Per	D1:Se1:0.81;Sp1:0.50;Az1:0.73 Se2:0.76;Sp2:1.00;Az2:0.87	D1:Se1:0.50;Sp1:1.00;Az1:0.86 Se2:0.42;Sp2:0.92;Az2:0.63		Histology
					D3:Se1:0.55;Sp1:0.81;Az1:0.65 Se2:0.58;Sp2:0.89;Az2:0.84	D3:Se1:0.50;Sp1:0.73;Az1:0.64 Se2:1.00;Sp2:0.81;Az2:0.89		
Jablonski-Momeni 2011a [28]	2	99	In vitro	Per	D1:Se1:0.81;Sp1:0.40;Az1:0.77 Se2:0.86;Sp2:0.32;Az2:0.75	N/A		Histology
					D3:Se1:0.09;Sp1:0.97;Az1:0.81 Se2:0.04;Sp2:0.99;Az2:0.77			
Jablonski-Momeni 2011b [9]	1	100	In vitro	Per	N/A	D1:Se:0.82;Sp:0.48;Az:0.746		Histology
						D3:Se:0.54;Sp:0.89;Az:0.786		
Rodrigues 2011 [10]	2	97	In vitro	Per	D1;Se:0.750;Sp:0.706;Az:0.760	D1:Se:0.700;Sp:0.765;Az:0.720		Histology
					D3:Se:0.963;Sp:0.700;Az:0.890	D3:Se:0.630;Sp:0.886;Az:0.850		
Aktan 2012 [11]	2	129	In vitro	Per	N/A	D1:Se1:0.65;Sp1:0.97;Az1:0.769 Se2:0.65;Sp2:0.97;Az2:0.755		Histology
						D3:Se1:0.33;Sp1:0.60;Az1:0.729 Se2:0.43;Sp2:0.49;Az2:0.697		
Diniz 2012 [12]	1	105	In vivo	Per	D1:Se:0.74;Sp:0.80;Az:0.79	D1:Se:0.89;Sp:0.80;Az:0.95		Histology
					D3:Se:0.85;Sp:0.49;Az:0.72	D3:Se:0.85;Sp:0.71;Az:0.79		
Jablonski-Momeni 2012a [13]	2	84	In vitro	Per	N/A	D1:Se:0.690;Sp:0.923;Az:0.88		Histology
						D3:Se:0.525;Sp:0.909;Az:0.88		
Jablonski-Momeni 2012b [14]	2	82	In vitro	Per	D1:Se1:0.65;Sp1:0.60; Az1:0.71 Se2:0.73;Sp2:0.50;Az2:0.96	D1:Se1:0.77;Sp1:0.40;Az1:0.72 Se2:0.81;Sp2:0.60;Az2:0.67		Histology
					D3:Se1:0.57;Sp1:0.66;Az1:0.65 Se2:0.86;Sp2:0.55;Az2:0.91	D3:Se1:0.71;Sp1:0.55;Az1:0.58 Se2:0.71;Sp2:0.52;Az2:0.91		
Rechmann 2012 [3]	1	433	In vivo	Per	N/A	Se:0.87;Sp:0.66;Az:0.87		Cutoff points
Seremidi 2012 [15]	4	107	In vitro	Per	D1:Se:0.568;Sp:0.586;Az:0.577	D1:Se:0.432;Sp:0.814;Az:0.623		Histology
					D3;Se:0.876;Sp:0.125;Az:0.550	D3:Se:0.795;Sp:0.750;Az:0.773		
Achilleos 2013 [4]	2	38	In vitro	Per	Se1:0.97;Sp1:0.50;Az1:0.486 Se2:0.97;Sp2:0.50;Az2:0.50	Se1:0.75;Sp1:0.50;Az1:0.583 Se2:0.66;Sp2:0.50;Az2:0.375		Histology
Jablonski-Momeni 2013 [29]	2	101	In vitro	Per	D1:Se1:0.818;Sp1:0.933;Az1:0.93 Se2:0.870;Sp2:1.000;Az2:0.96	N/A		Histology
					D3:Se1:0.907;Sp1:0.776;Az1:0.93 Se2:0.953;Sp2:0.714;Az2:0.91			
Teo 2014 [16]	2	102	In vivo/ In vitro	Pri	N/A	VivoD1:Se:0.87; Sp:0.44;Az:0.83	VitroD1:Se:0.86; Sp:0.92;Az:0.92	Histology
						VivoD3:Se:0.95; Sp:0.64;Az:0.93	VitroD3:Se:0.98; Sp:0.77;Az:0.93	
Jablonski-Momeni 2014 [5]	1	306	In vivo	Per	D1:Se:0.922;Sp:0.460;Az:0.82	N/A		*
Sinanoglu 2014 [18]	2	217	In Vivo	Per	N/A	Se1:0.89;Sp1:0.75;Az1:0.6407 Se2:0.72;Sp2:0.86;Az2:0.5558		Histology
Bussaneli 2015 [17]	2	102	In vitro	Per	N/A	Se:0.662;Sp:0.821;Az:0.759		Histology
Melo 2015 [19]	1	32	In vivo	Per	Se:0.964;Sp:1.000;Az:0.969	Se:1.000;Sp:0.750;Az:0.969		Histology
Ozturk 2015 [20]	2	121	In vitro	Per	N/A	Se1:0.86;Sp1:0.69;Az1:0.77 Se2:0.86;Sp2:0.71;Az2:0.78		Histology
Theocharopoulou 2015 [21]	1	37	In vivo	Pri/Per	N/A	Se:0.62;Sp:0.81;Az:N/A		ICDAS
Castilho 2016 [22]	1	43	In vivo	Per	N/A	Se:0.85;Sp:0.25;Az:0.55		Histology
Novaes 2016 [23]	2	99	In vitro	Pri	D1:Se1:0.77;Sp1:0.82;Az1:0.79 Se2:0.80;Sp2:0.61;Az2:0.74	D1:Se1:0.96;Sp1:0.21;Az1:0.71 Se2:0.76;Sp2:0.67;Az2:0.73		Histology
					D3:Se1:0.52;Sp1:0.97;Az1:0.86 Se2:0.68;Sp2:0.99;Az2:0.91	D3:Se1:0.60; Sp1:0.82; Az1:0.76 Se2:0.56;Sp2:0.95;Az2:0.85		
Peycheva 2016 [24]	2	45	In vitro	Per	N/A	D3:Se:0.92;Sp:0.69;Az:N/A		Histology
Iranzo-Cortés 2017 [25]	2	65	In vitro	Per	N/A	Se:0.85;Sp:0.53;Az:0.73		Histology
Kockanat 2017 [26]	2	120	In vivo/In vitro	Pri	N/A	VivoD1:Se1:0.89; Sp1:1.0;Az1:0.95 Se2:0.89;Sp2:0.98; Az2:0.94	VitroD1:Se1:0.92;Sp1:0.94;Az1:0.90 Se2:0.88;Sp2:0.94; Az2:0.88	Histology
						VivoD3:Se1:0.81; Sp1:0.97;Az1:0.92 Se2:0.81;Sp2:0.97; Az2:0.90	VitroD3:Se1:0.78; Sp1:0.97;Az1:0.95 Se2:0.78;Sp2:0.97; Az2:0.94	
Melo 2017 [27]	1	302	In vivo	Per	Se:0.929;Sp:0.958;Az:0.965	Se:0.924;Sp:0.927;Az:0.954		Histology
Iranzo-Cortés 2018 [30]	1	65	In vitro	Per	Se1:0.70;Sp1:0.81;Az1:0.76 Se2:0.82;Sp2:0.62;Az2:0.72	N/A		Histology

**Table 2:** Quality assessment of the studies following QUADAS-2 assessment tool. Risk: Low = √; Uncertain = ¿?; High = X.

Study	Risk of Bias				Concerns Regarding Applicability		
	Patient Selection	Index Test	Reference Standard	Flow and Timing	Patient Selection	Index Test	Reference Standard
Rodrigues 2008 [7]	¿?	√	√	√	¿?	√	√
Diniz 2011 [8]	√	√	√	√	√	√	√
Jablonski-Momeni 2011a [28]	¿?	√	√	√	¿?	√	√
Jablonski-Momeni 2011b [9]	¿?	√	√	√	¿?	√	√
Rodrigues 2011 [10]	¿?	√	√	√	¿?	√	√
Aktan 2012 [11]	¿?	√	√	√	¿?	√	√
Diniz 2012 [12]	¿?	¿?	√	√	¿?	¿?	√
Jablonski-Momeni 2012a [13]	√	√	√	√	√	√	√
Jablonski-Momeni 2012b [14]	¿?	√	√	√	¿?	√	√
Rechmann 2012 [3]	√	√	X	√	√	√	X
Seremidi 2012 [15]	¿?	√	√	√	¿?	√	√
Achilleos 2013 [4]	√	√	√	√	√	√	√
Jablonski-Momeni 2013 [29]	¿?	√	√	√	¿?	√	√
Teo 2014 [16]	¿?	√	√	√	¿?	√	√
Jablonski-Momeni 2014 [5]	¿?	√	X	√	¿?	√	X
Sinanoglu 2014 [18]	¿?	√	√	√	¿?	√	√
Bussaneli 2015 [17]	¿?	√	√	√	¿?	√	√
Melo 2015 [27]	¿?	√	√	√	¿?	√	√
Ozturk 2015 [20]	¿?	√	√	√	¿?	√	√
Theocharopoulou 2015 [21]	X	√	X	√	√	√	X
Castilho 2016 [22]	X	√	√	√	√	√	√
Novaes 2016 [23]	¿?	√	√	√	¿?	√	√
Peycheva 2016 [24]	¿?	√	√	√	¿?	√	√
Iranzo-Cortés 2017 [25]	√	√	√	√	¿?	√	√
Kockanat 2017 [26]	√	√	√	√	√	√	√
Melo 2017 [27]	¿?	√	¿?	√	¿?	√	¿?
Iranzo-Cortés 2018 [30]	√	√	√	√	¿?	√	√

**Table 3:** Sensitivity, specificity and AUC for all studies combined and for subgroups (CI-95%).

	DIAGNOdent			VistaProof		
	All	Subgroup		All	Subgroup	
		In Vitro	In Vivo		In Vitro	In Vivo
Sensitivity	0.772 (0.703–0.829)	0.714 (0.626–0.788)	0.852 (0.780–0.903)	0.813 (0.681–0.899)	0.747 (0.570–0.868)	0.912 (0.868–0.942)
Specificity	0.754 (0.695–0.805)	0.752 (0.680–0.813)	0.758 (0.639–0.847)	0.754 (0.620–0.852)	0.739 (0.581–0.852)	0.807 (0.476–0.951)
AUC	0.810 (0.758–0.853)	0.781 (0.726–0.827)	0.861 (0.752–0.927)	0.798 (0.719–0.860)	0.755 (0.662–0.829)	0.892 (0.748–0.959)

## Supplementary Materials

The following are available online at https://www.mdpi.com/2077-0383/9/1/20/s1, Figure S1: Forest Plot One Study Removed for Sensitivity of DIAGNOdent; Figure S2: Forest Plot One Study Removed for Specificity of DIAGNOdent; Figure S3: Forest Plot One Study Removed for AUC of DIAGNOdent; Figure S4: Forest Plot One Study Removed for Sensitivity of VistaProof; Figure S5: Forest Plot One Study Removed for Specificity of VistaProof; Figure S6: Forest Plot One Study Removed for AUC of VistaProof

## References

[1] Featherstone J.D. (2008). Dental caries: A dynamic disease process. Aust. Dent. J..

[2] Kuhnisch J., Berger S., Goddon I., Senkel H., Pitts N., Heinrich-Weltzien R. (2008). Occlusal caries detection in permanent molars according to WHO basic methods, ICDAS II and laser fluorescence measurements. Community Dent. Oral Epidemiol..

[3] Rechmann P., Charland D., Rechmann B.M., Featherstone J.D. (2012). Performance of laser fluorescence devices and visual examination for the detection of occlusal caries in permanent molars. J. Biomed. Opt..

[4] Achilleos E.E., Rahiotis C., Kakaboura A., Vougiouklakis G. (2013). Evaluation of a new fluorescence-based device in the detection of incipient occlusal caries lesions. Lasers Med. Sci..

[5] Jablonski-Momeni A., Heinzel-Gutenbrunner M., Klein S.M. (2014). In vivo performance of the VistaProof fluorescence-based camera for detection of occlusal lesions. Clin. Oral Investig..

[6] Whiting P.F., Rutjes A.W.S., Westwood M.E., Mallett S., Deeks J.J., Reitsma J.B., Leeflang M.M.G., Sterne J.A.C., Bossuyt P.M.M., Altman D. (2011). QUADAS-2: A revised tool for the quality assessment of diagnostic accuracy studies. Ann. Intern. Med..

[7] Rodrigues J.A., Hug I., Diniz M.B., Lussi A. (2008). Performance of fluorescence methods, radiographic examination and ICDAS II on occlusal surfaces in vitro. Caries Res..

[8] Diniz M.B., Sciasci P., Rodrigues J.A., Lussi A., Cordeiro R.C.L. (2011). Influence of different professional prophylactic methods on fluorescence measurements for detection of occlusal caries. Caries Res..

[9] Jablonski-Momeni A., Ricketts D.N., Rolfsen S., Stoll R., Heinzel-Gutenbrunner M., Stachniss V., Pieper K. (2011). Performance of laser fluorescence at tooth surface and histological section. Lasers Med. Sci..

[10] Rodrigues J., Hug I., Neuhaus K.W., Lussi A. (2011). Light-emitting diode and laser fluorescence-based devices in detecting occlusal caries. J. Biomed. Opt..

[11] Aktan A., Cebe M., Çiftçi M., Şirin Karaarslan E. (2012). A novel LED-based device for occlusal caries detection. Lasers Med. Sci..

[12] Diniz M.B., Boldieri T., Rodrigues J.A., Santos-Pinto L., Lussi A., Cordeiro R.C. (2012). The performance of conventional and fluorescence-based methods for occlusal caries detection: An in vivo study with histologic validation. J. Am. Dent. Assoc..

[13] Jablonski-Momeni A., Stucke J., Steinberg T., Heinzel-Gutenbrunner M. (2012). Use of ICDAS-II, Fluorescence-Based Methods, and Radiography in Detection and Treatment Decision of Occlusal Caries Lesions: An In Vitro Study. Int. J. Dent..

[14] Jablonski-Momeni A., Rosen S., Schipper H., Stoll R., Roggendorf M., Heinzel-Gutenbrunner M., Stachniss V., Pieper K. (2012). Impact of measuring multiple or single occlusal lesions on estimates of diagnostic accuracy using fluorescence methods. Lasers Med. Sci..

[15] Seremidi K., Lagouvardos P., Kavvadia K. (2012). Comparative in vitro validation of VistaProof and DIAGNOdent pen for occlusal caries detection in permanent teeth. Oper. Dent..

[16] Teo T.K., Ashley P.F., Louca C. (2014). An in vivo and in vitro investigation of the use of ICDAS, DIAGNOdent pen and CarieScan PRO for the detection and assessment of occlusal caries in primary molar teeth. Clin. Oral Investig..

[17] Bussaneli D.G., Restrepo M., Boldieri T., Pretel H., Mancini M.W., Santos-Pinto L., Cordeiro R.C. (2015). Assessment of a new infrared laser transillumination technology (808 nm) for the detection of occlusal caries-an in vitro study. Lasers Med. Sci..

[18] Sinanoglu A., Ozturk E., Ozel E. (2014). Diagnosis of Occlusal Caries Using Laser Fluorescence Versus Conventional Methods in Permanent Posterior Teeth: A Clinical Study. Photomed. Laser Surg..

[19] Melo M., Pascual A., Camps I., Del Campo Á. (2015). In vivo study of different methods for diagnosing pit and fissure caries. J. Clin. Exp. Dent..

[20] Ozturk E., Sinanoglu A. (2015). Histological validation of cone-beam computed tomography versus laser fluorescence and conventional diagnostic methods for occlusal caries detection. Photomed. Laser Surg..

[21] Theocharopoulou A., Lagerweij M.D., van Strijp A.J. (2015). Use of the ICDAS system and two fluorescence-based intraoral devices for examination of occlusal surfaces. Eur. J. Paediatr. Dent..

[22] Castilho L.S., Cotta F.V., Bueno A.C., Moreira A.N., Ferreira E.F., Magalhaes C.S. (2016). Validation of DIAGNOdent laser fluorescence and the International Caries Detection and Assessment System (ICDAS) in diagnosis of occlusal caries in permanent teeth: An in vivo study. Eur. J. Oral Sci..

[23] Novaes T.F., Moriyama C.M., De Benedetto M.S., Kohara E.K., Braga M.M., Mendes F.M. (2016). Performance of fluorescence-based methods for detecting and quantifying smooth-surface caries lesions in primary teeth: An in vitro study. Int. J. Paediatr. Dent..

[24] Peycheva K., Boteva E. (2016). A Comparison of Different Methods for Fissure Caries Detection. Acta Med. Bulg..

[25] Iranzo-Cortes J.E., Terzic S., Montiel-Company J.M., Almerich-Silla J.M. (2017). Diagnostic validity of ICDAS and DIAGNOdent combined: An in vitro study in pre-cavitated lesions. Lasers Med. Sci..

[26] Kockanat A., Unal M. (2017). In vivo and in vitro comparison of ICDAS II, DIAGNOdent pen, CarieScan PRO and SoproLife camera for occlusal caries detection in primary molar teeth. Eur. J. Paediatr. Dent..

[27] Melo M., Pascual A., Camps I., del Campo Á., Ata-Ali J. (2016). Caries diagnosis using light fluorescence devices in comparison with traditional visual and tactile evaluation: A prospective study in 152 patients. Odontology.

[28] Jablonski-Momeni A., Schipper H., Rosen S., Heinzel-Gutenbrunner M., Roggendorf M., Stoll R., Stachniss V., Pieper K. (2011). Performance of a fluorescence camera for detection of occlusal caries in vitro. Odontology.

[29] Jablonski-Momeni A., Liebegall F., Stoll R., Heinzel-Gutenbrunner M., Pieper K. (2013). Performance of a new fluorescence camera for detection of occlusal caries in vitro. Lasers Med. Sci..

[30] Iranzo-Cortes J.E., Almarche-Tarazona M., Montiel-Company J.M., Almerich-Silla J.M. (2018). Diagnostic validity of ICDAS II, VistaProof and a combination of these two methods. An in vitro study in pre-cavitated lesions. Lasers Surg. Med..

[31] Gomez J., Tellez M., Pretty I.A., Ellwood R.P., Ismail A.I. (2013). Non-cavitated carious lesions detection methods: A systematic review. Community Dent. Oral Epidemiol..

