# Supplementary figures and images for: Use of DIAGNOdent and VistaProof in diagnostic of Pre-Cavitated Caries Lesions—A Systematic Review and Meta-Analysis
Source: J Clin Med. 2019 Dec 19;9(1):20. doi: 10.3390/jcm9010020 (PMC7019252; doi:10.3390/jcm9010020)

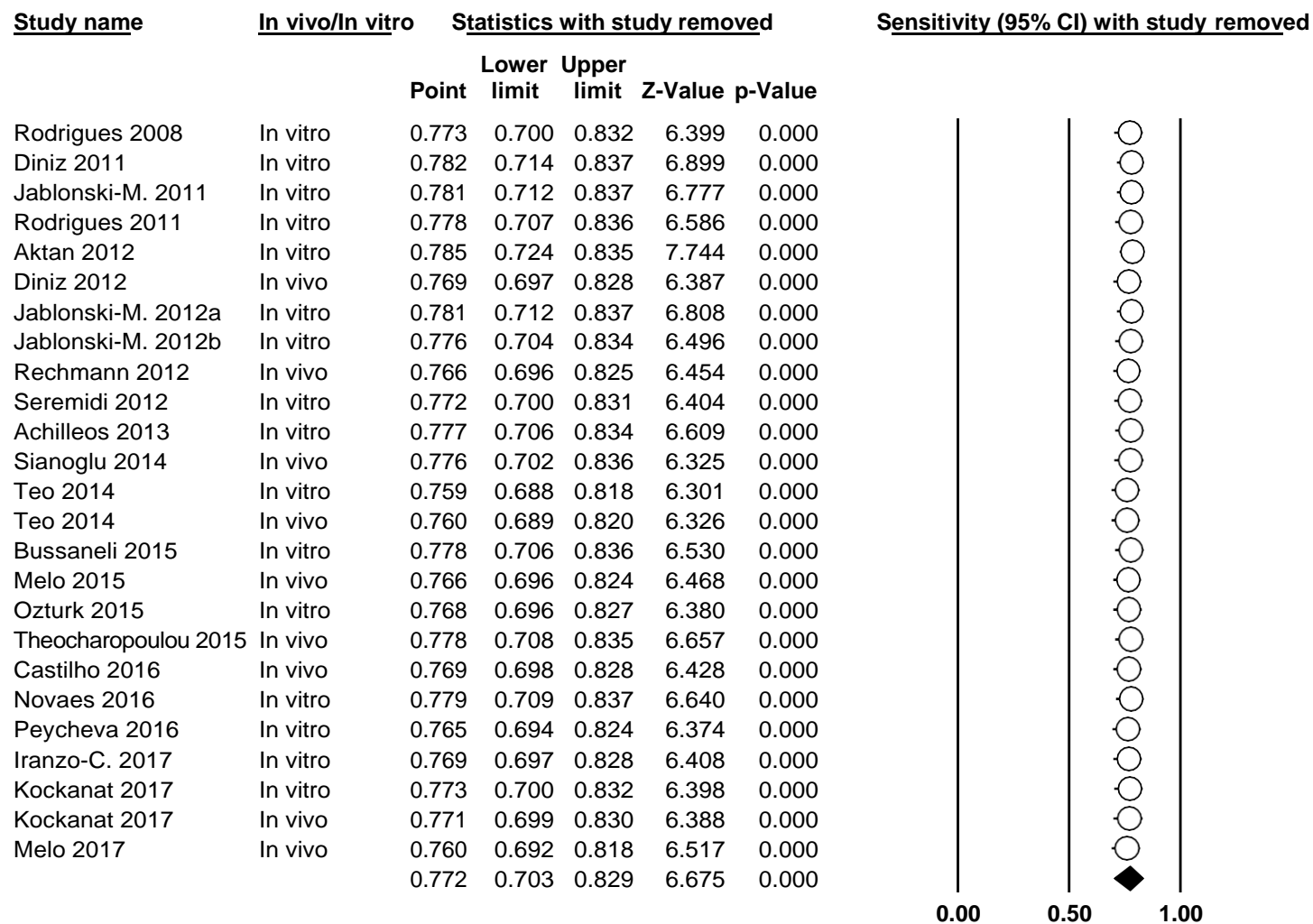

Supplement: Supplementary file 1 [file jcm-09-00020-s001.zip › Supplementary_figures_2.0/Figure_S1b.pdf]

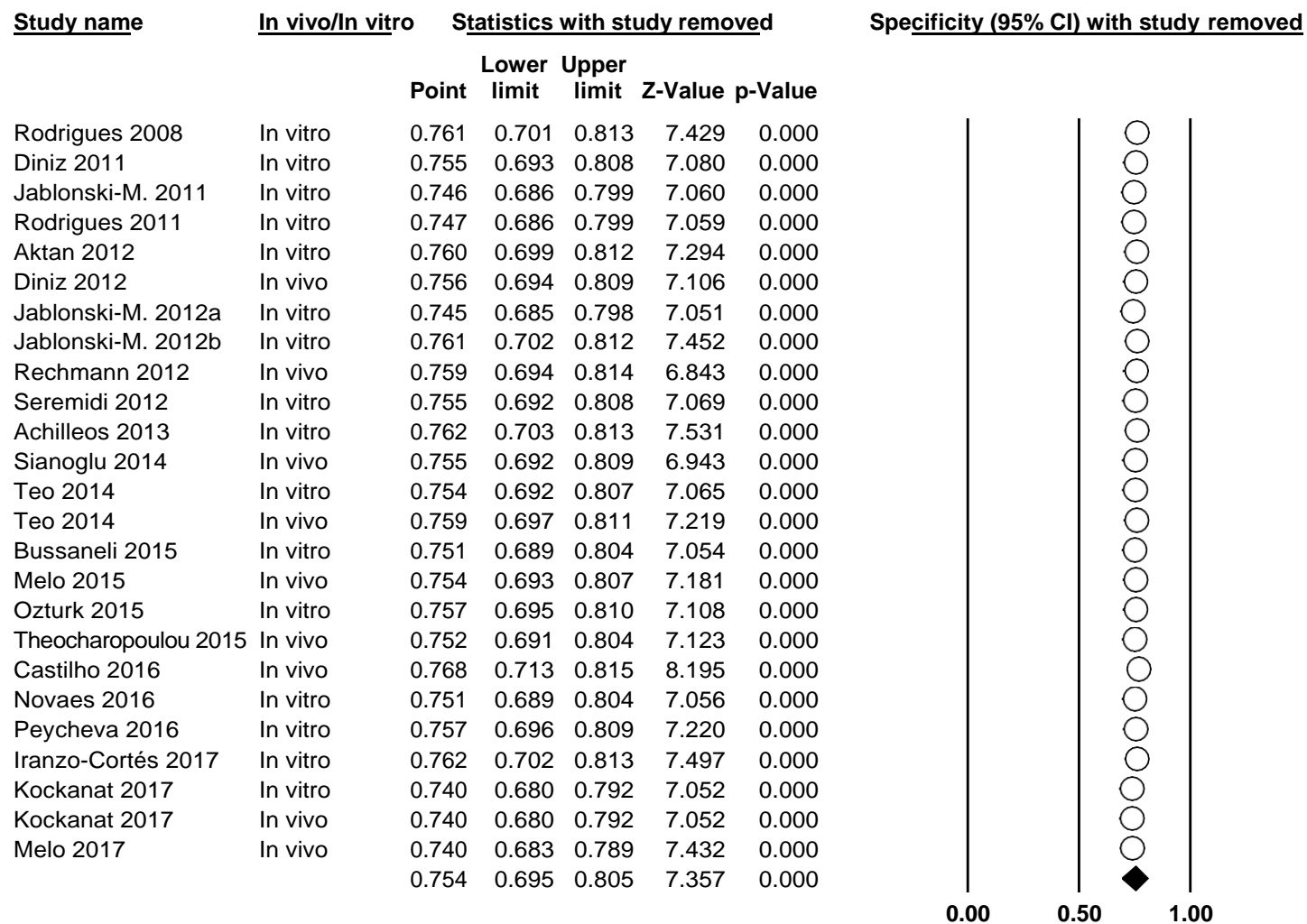

Supplement: Supplementary file 1 [file jcm-09-00020-s001.zip › Supplementary_figures_2.0/Figure_S2b.pdf]

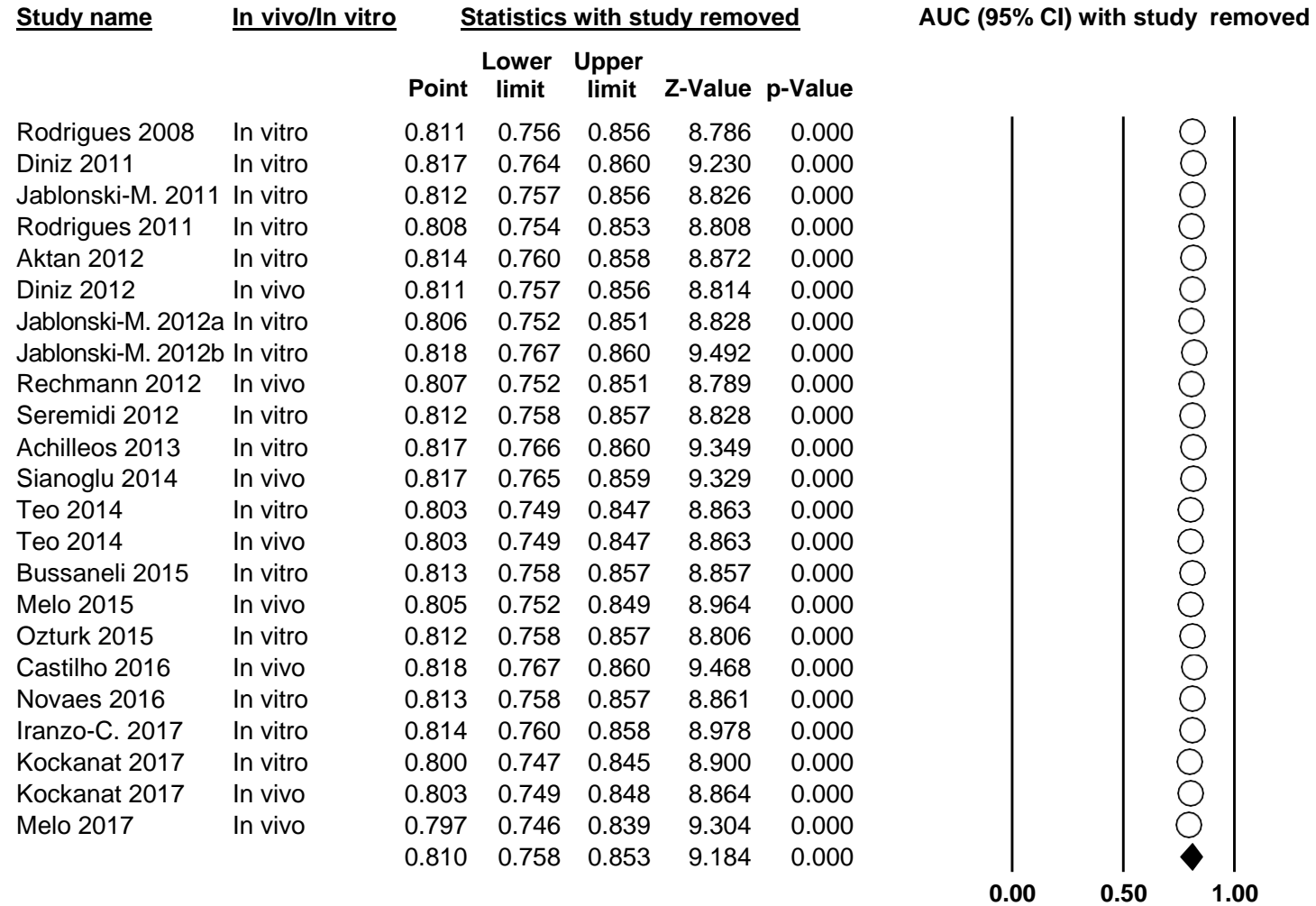

Supplement: Supplementary file 1 [file jcm-09-00020-s001.zip › Supplementary_figures_2.0/Figure_S3b.pdf]

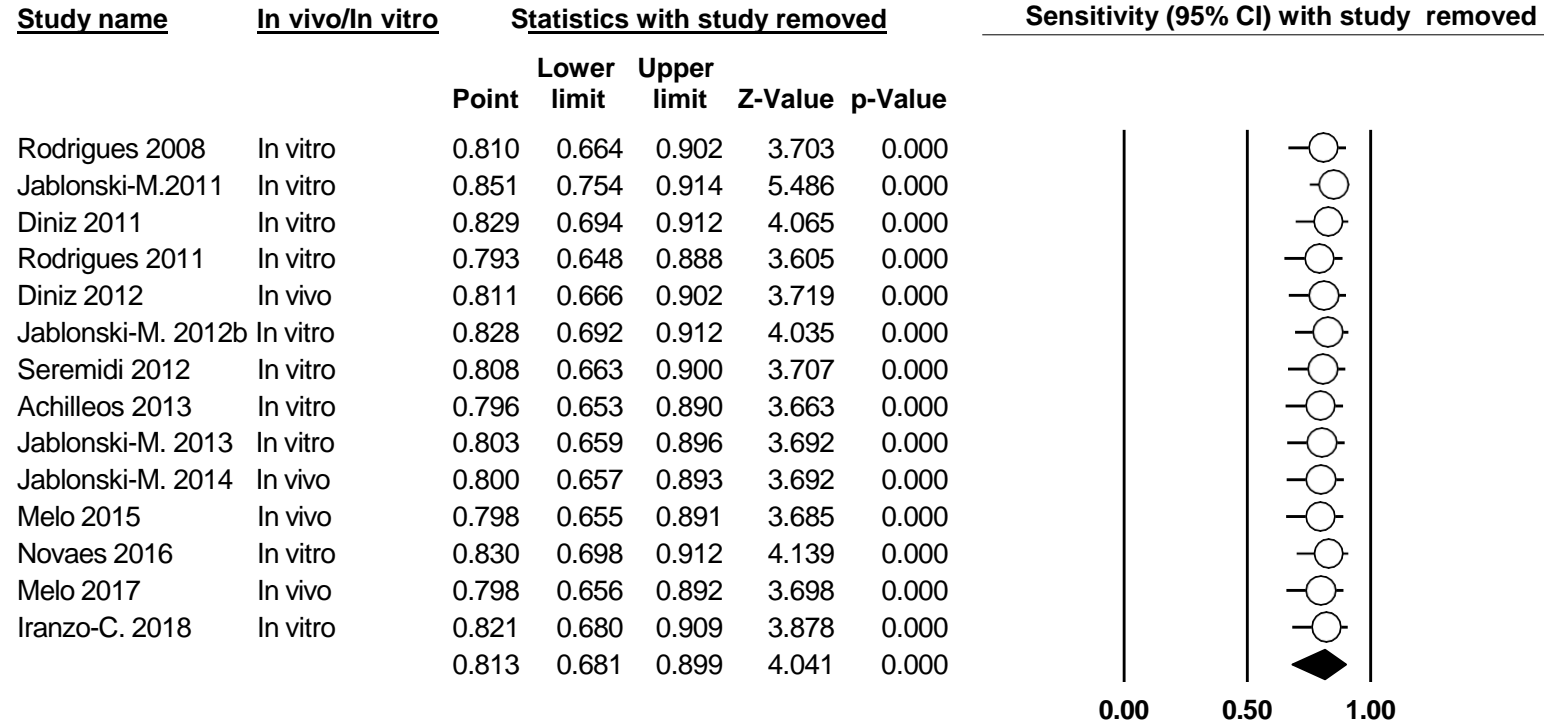

Supplement: Supplementary file 1 [file jcm-09-00020-s001.zip › Supplementary_figures_2.0/Figure_S4b.pdf]

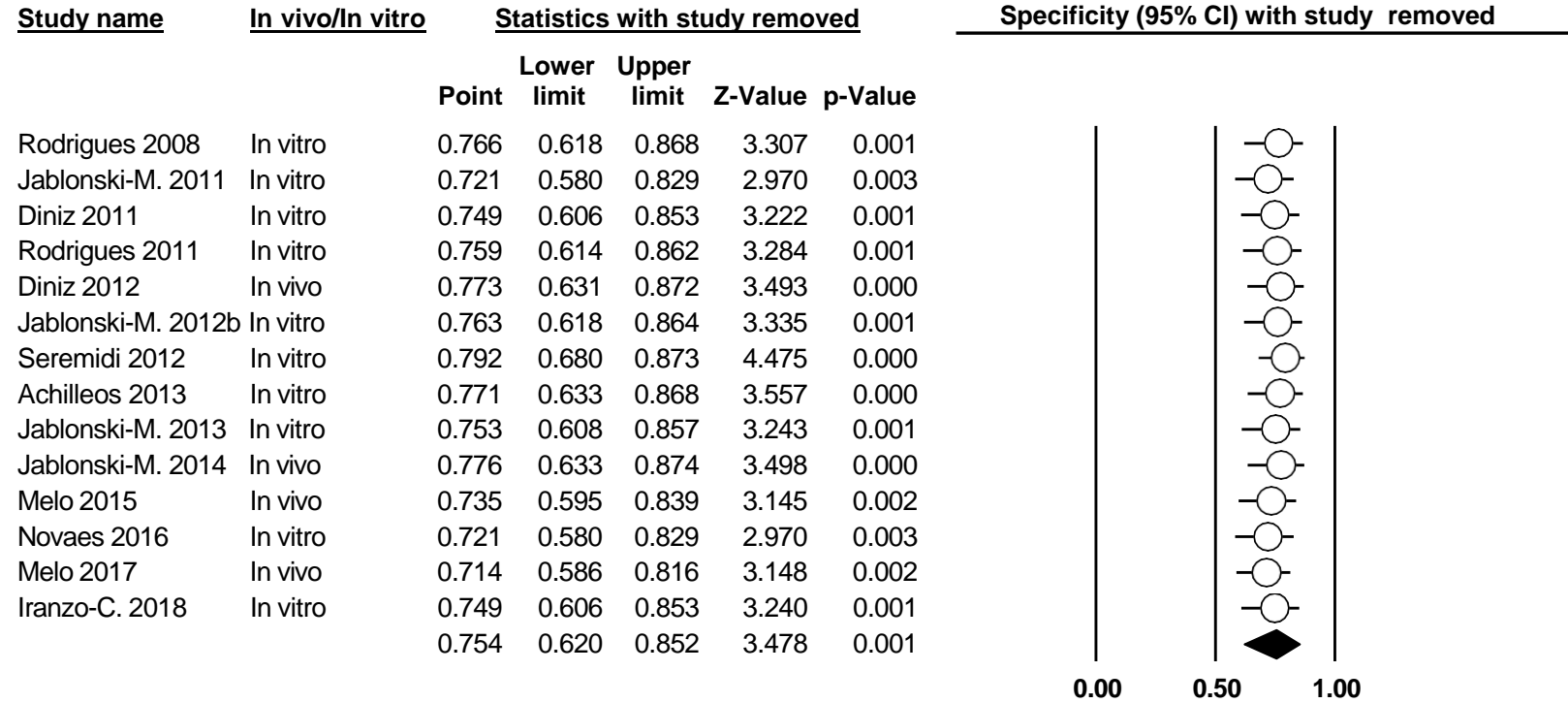

Supplement: Supplementary file 1 [file jcm-09-00020-s001.zip › Supplementary_figures_2.0/Figure_S5b.pdf]

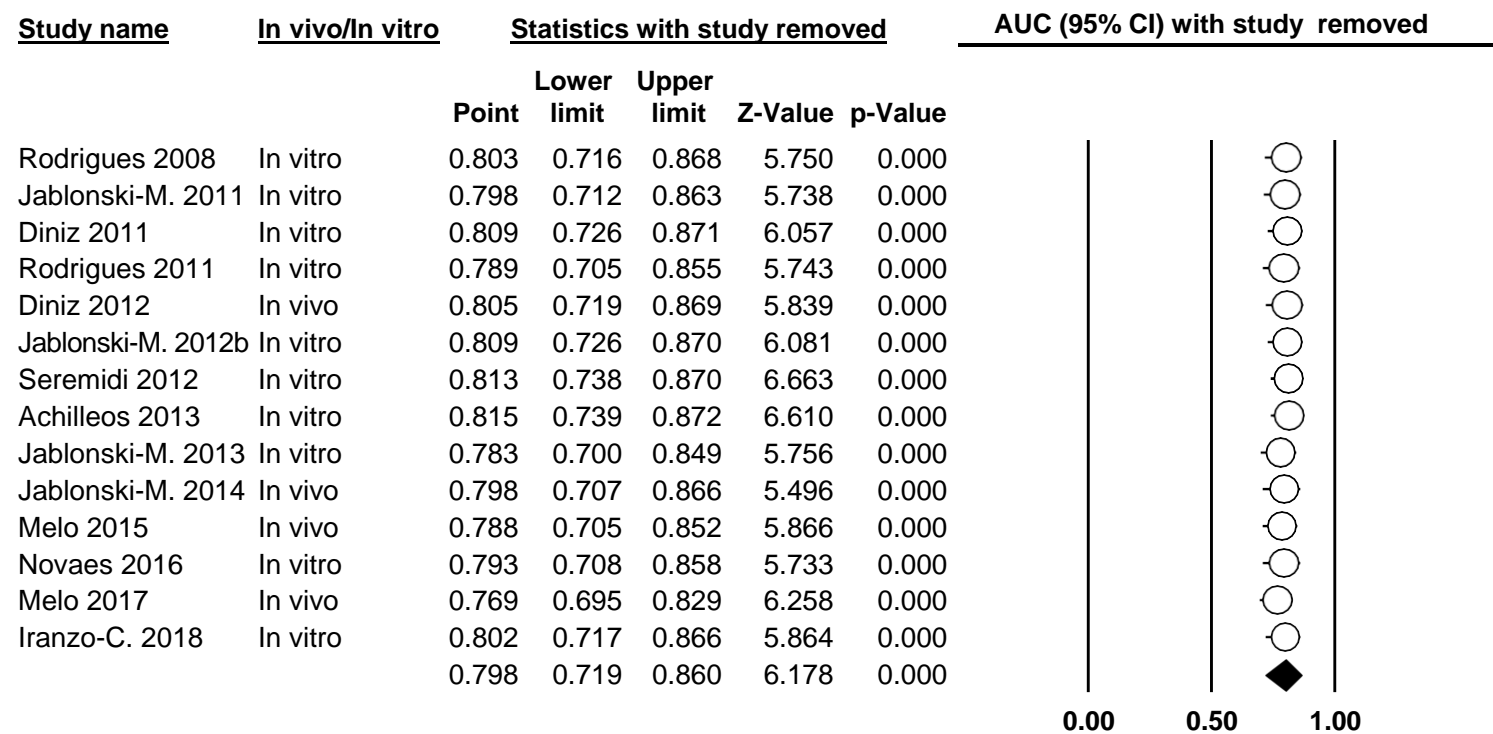

Supplement: Supplementary file 1 [file jcm-09-00020-s001.zip › Supplementary_figures_2.0/Figure_S6b.pdf]
